# Trends and incidence rates of neurodevelopmental disorders in Danish children and adolescents 2000–2024: Short title: neurodevelopmental disorders in children and adolescents

**DOI:** 10.1007/s00787-026-02981-0

**Published:** 2026-02-10

**Authors:** M. Bliddal, E. B. Gram, H. Sonne, J. R. Krumborg, K. M. Lundgaard, R. Wesselhoeft, H. Kildegaard

**Affiliations:** 1 Clinical Pharmacology, Pharmacy and Environmental Medicine, Department of Public Health, University of Southern Denmark, Odense, Denmark; 2 Gynecology and Obstetrics, Department of Clinical Research, University of Southern Denmark, Odense, Denmark; 3 Department of Gynecology and Obstetrics, Odense University Hospital, Odense, Denmark; 4 Research Unit of Clinical Pharmacology, Department of Clinical Research, University of Southern Denmark, Odense, Denmark; 5 Child and Adolescent Psychiatry Southern Denmark, Mental Health Services in the Region of Southern Denmark, Odense, Denmark; 6 Centre for Pharmacoepidemiology, Department of Medicine, Karolinska Institutet, Stockholm, Sweden; 7 Mary Elizabeth’s Hospital, University Hospital Copenhagen, Rigshospitalet, Denmark

**Keywords:** Neurodevelopmental disorders, Autism spectrum disorders, Attention-Deficit/Hyperactivity disorder, Incidence, Sex differences

## Abstract

**Background:**

Autism Spectrum Disorder (ASD) and Attention-Deficit/Hyperactivity Disorder (ADHD) are prevalent neurodevelopmental disorders with long-term consequences. Recognition is essential but often delayed, particularly in females. Many cases are not captured in national registers, though many ADHD are often identifiable through prescriptions. We examined age- and sex-specific incidence and temporal trends in ASD and ADHD diagnosis in Denmark from 2000 to 2024 and assessed how including prescription data affects ADHD estimates.

**Methods:**

A cohort of all children residing in Denmark from 2000 to 2024 (*n* = 2.8 million) was followed until age 18 or December 31, 2024. Using nationwide registers, we identified diagnoses of ASD and ADHD. For ADHD, we extended the definition to include ADHD-specific prescriptions. We estimated age- and sex-specific incidence rates (IRs), cumulative incidence at each 1-year age point, and trends by birth cohort.

**Results:**

Among 2.8 million children, 60,432 were diagnosed with ASD and 102,051 with ADHD. Incidence rates increased for both conditions, with a steeper rise among females. By age 18, cumulative incidence was 6.5% (95% confidence intervals (CI) 6.5–6.6) in males and 3.9% (95% CI 3.8-4.0) in females for ASD, and 9.8% (95% CI 9.8–9.9) and 7.4% (95% CI 7.3–7.5) for ADHD.

Although rates remained higher in males, the sex gap narrowed substantially, particularly for ADHD. Including ADHD-specific prescriptions raised cumulative ADHD incidence by age 18 from 6.9% (95% CI 6.8–6.9) to 8.7% (95% CI 8.6–8.7).

**Conclusion:**

The incidence of ASD and ADHD has risen substantially in Denmark over the past two decades, most pronounced among girls, narrowing the historical sex gap. Incorporating prescription data increased ADHD incidence estimates notably, underscoring the importance of broader case definitions in epidemiological research.

**Supplementary Information:**

The online version contains supplementary material available at 10.1007/s00787-026-02981-0.

## Introduction

Neurodevelopmental disorders, including Autism Spectrum Disorder (ASD) and Attention-Deficit/Hyperactivity Disorder (ADHD), substantially impact the lives of children and adolescents, negatively affecting academic achievement and long-term well-being [1, 2]. These challenges are exacerbated when affected children do not receive timely recognition and support, underscoring the importance of early and accurate detection [1, 2]. Over the past two decades, the cumulative incidence of both ASD and ADHD has increased, accompanied by a trend towards earlier age at diagnosis [3–6]. Marked sex differences exist in the recognition and diagnosis of neurodevelopmental disorders, likely reflecting variation in symptom presentation [7, 8]. These differences may lead to disparities in access to treatment and long-term outcomes. Understanding sex-specific incidence is essential to inform public health strategies, prioritize resource allocation, and identify potential inequalities in mental health services for male and female youths.

In parallel with the rise in youths diagnosed with clinical neurodevelopmental disorders in recent years, the use of psychotropic medications has increased globally, particularly for managing ADHD [9–12]. Due to capacity constraints in hospital-based child and adolescent psychiatry, an increasing number of children are diagnosed and managed by private practicing psychiatric specialists [13]. However, such diagnoses are often not captured in national patient registers, leading to potential underestimation of ASD and ADHD incidence in register-based studies. This may distort incidence estimates and bias the reported age-of-diagnosis as a proxy for time of disease detection. Despite these developments, most existing studies rely exclusively on recorded diagnoses to estimate ADHD incidence. Supplementing this data with the use of ADHD-specific medications may improve case identification and reduce underestimation of clinically recognized cases.

Although several Danish studies have examined trends in neurodevelopmental disorders, recent Danish evidence is limited, as earlier studies cover older periods or the full population [3, 4]. Specifically, updated age- and sex-specific estimates for children and adolescents are lacking despite marked changes in diagnostic activity, particularly during and after the COVID-19 pandemic [5, 6, 14].

Several factors have been proposed to explain the increasing incidence and prevalence of clinically diagnosed neurodevelopmental disorders in Western countries, including changes in diagnostic practices, increased awareness, and improved recognition of sex-specific symptom presentations [14–16]. Some authors have further suggested that increasing societal complexity, pervasive media exposure, rising performance demands, and pandemic-related disruptions may contribute to greater help-seeking and diagnostic activity, particularly in adolescence [15, 17–19].

In this study, we aimed to assess age- and sex-specific incidence rates, cumulative incidences, and temporal trends of ASD and ADHD among children and adolescents in Denmark from 2000 to 2024. We further assessed the cumulative incidence by birth cohort to evaluate changes over time. Finally, we evaluated the impact of including both diagnostic codes and prescription data for ADHD-specific medications when assessing the incidence of ADHD.

## Methods

### Study population

In this nationwide cohort study, we included all individuals aged 0–17 years residing in Denmark between January 1, 2000, and December 31, 2024. All Danish residents are assigned a unique personal identification number at birth or upon first immigration. This is recorded in the Danish Civil Registration System along with key demographic information, including date of birth, sex, and continuous updates on vital status and migration [20]. Using this unique identifier, we performed individual-level linkage of national registers to obtain data on hospital diagnoses and medication use.

### Neurodevelopmental disorders

Information on neurodevelopmental disorders was obtained from the Danish National Patient Register and Danish National Prescription Register [21, 22]. The Danish National Patient Register holds information on all in- and outpatient hospital contacts since 1995. It includes data on date of contact and diagnoses coded according to the International Classification of Diseases, version 10 (ICD-10). The Danish National Prescription Register holds data on all redeemed prescriptions from pharmacies in Denmark since 1995, with documented high validity and completeness. All drugs are coded according to the Anatomical Therapeutic Chemical (ATC) Classification system [23], and the register informs on date of redemption, quantity, and specialty of the provider [22].

Autism spectrum disorders were defined by the presence of at least one ICD-10 code in the National Patient Register of either F84.0, F84.1, F84.5, F84.8, or F84.9. Only diagnoses recorded at age 1 year or older were considered valid [24]. Attention-deficit/hyperactivity disorder was defined as either an ICD-10 diagnosis of F90.x or F98.8, or at least one redeemed prescription of an ADHD-specific medication. In Denmark, ADHD is classified and diagnosed under ICD-10 as hyperkinetic disorders (code F90.x), or as other specified behavioral and emotional disorders with onset in childhood or adolescence (F98.8). The latter is used to capture cases of attention deficit disorder without hyperactivity. Attention-deficit/hyperactivity disorder specific medications included methylphenidate (ATC: N06BA04), atomoxetine (N06BA09), dexamphetamine (N06BA02), lisdexamphetamine (N06BA12), and guanfacine (C02AC02). Only diagnoses and prescriptions recorded at age 3 years or older were included [25]. The stated indication is available in the prescription register, and in 2024 more than 98% of ADHD-specific prescriptions were issued for ADHD or attention difficulties.

Data on hospital diagnoses and redeemed prescriptions were available through December 31, 2024, which defined the end of the study period.

### Statistical analyses

First, we estimated the annual incidence rates (IRs) of ASD and ADHD per 10,000 individuals, overall and stratified by sex. The incidence rate was calculated as the number of new cases each year divided by the total population as of July 1 of the respective year, excluding individuals with a prior record of the disorder. For ASD, the denominator included individuals aged 1–18 years; for ADHD, it included individuals aged 3–18 years. The date of incidence was defined as the earliest date of either a first diagnosis record or a redeemed prescription.

Second, we estimated age-specific IRs and cumulative incidence proportions of ASD and ADHD. The cumulative incidence was calculated as the probability per 100 individuals in the population of being diagnosed before a given age, stratified by sex. Children were followed from birth until outcome, age 18, emigration, or end of follow-up, whichever came first. In sensitivity analyses, we restricted definitions of ASD and ADHD to increase validity. For ASD, we required at least two registered ICD-10 codes indicating more than one contact related to a given challenge. For ADHD, we required at least two registered ICD-10 codes, or at least two redeemed prescriptions, or one diagnosis code and one redeemed prescription.

Third, to examine changes in the cumulative incidence and age at diagnosis over time, we estimated sex-specific cumulative incidences of ASD and ADHD across four consecutive 5-year birth cohorts: 2000–2004, 2005–2009, 2010–2014, and 2015–2019. The youngest cohort (2020–2023) was excluded due to limited follow-up time.

Fourth, we examined how inclusion of redeemed prescriptions for ADHD-specific medications in the definition of ADHD affected the estimated cumulative incidence. To do this, we classified ADHD cases into two groups: those with an incident recorded ADHD diagnosis irrespective of medication use, and those with incident prescriptions only and no recorded diagnosis. Individuals with a prescription who received an ADHD diagnosis within 365 days were assigned to the first group, while the second group included only those without a diagnosis before or in the 365 days following the first prescription, to reduce misclassification due to delayed diagnosis. This analysis conditioned on the study populations being born in Denmark, and children were followed from birth until outcome, age 18, emigration, or end of follow-up whichever came first.

Finally, to assess potential changes in diagnostic and treatment patterns over time, we also calculated the annual proportion of ADHD cases identified solely through incident ADHD medication use, applying the same 365-day classification criteria as described above. For this analysis, the denominator included all individuals with either an incident prescription for ADHD-specific medication or an incident ADHD diagnosis within each calendar year.

### Ethics

The study was approved by the Danish Data Protection Agency through institutional registration at University of Southern Denmark (registration number 11.106). In accordance with Danish law, ethical approval and informed consent are not required for register-based research.

## Results

The study cohort included 2.8 million children residing in Denmark between 2000 and 2024, with follow-up until December 31, 2024. Of the total study population, 49% were female and 51% were male. A total of 60,432 individuals were identified with ASD (69% males) and 102,051 with ADHD (64% males) before age 18 years, with cumulative incidence at age 18 of 5.3% (95% confidence interval (CI) 5.2–5.3) for ASD and 8.7% (95%CI 8.6-8-7) for ADHD.

**Fig. 1:**
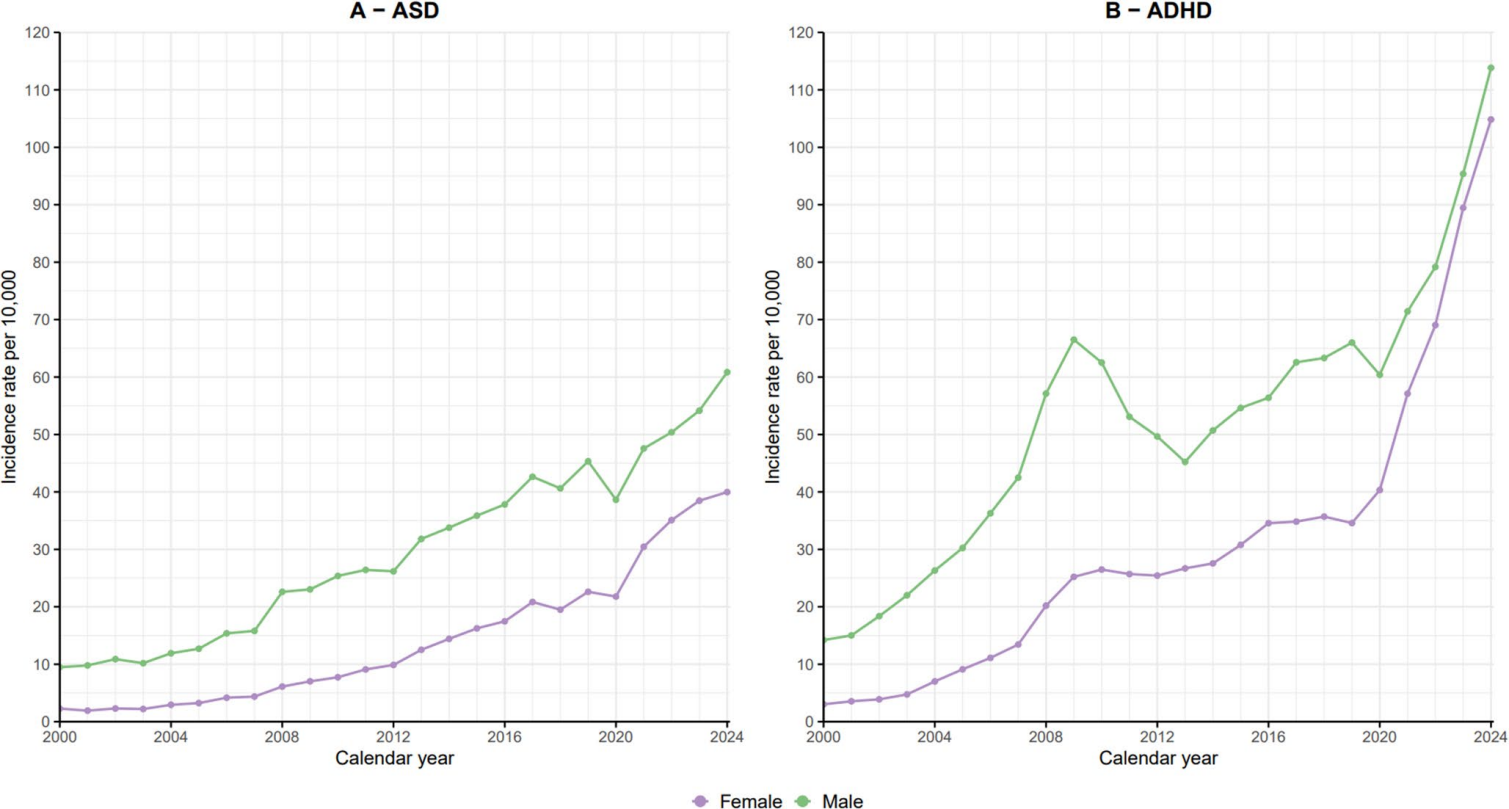
Incidence rates of (**A**) Autism Spectrum Disorders (ASD) and (**B**) Attention-Deficit/Hyperactivity Disorder (ADHD) in Danish children aged 0–17 years per 10,000 person years from 2000 to 2024

The IRs of ASD increased steadily across the study period for both sexes, with a transient decline observed in 2020 (Fig. 1A). Among males, ASD incidence rose from 9.5 per 10,000 in 2000 to 61 per 10,000 in 2024; for females, the corresponding increase was from 2.3 to 40 per 10,000. ADHD IRs increased from 14 to 66 per 10,000 in males and from 3.0 to 35 per 10,000 in females during 2000–2019, with a pronounced peak in incidence among males in 2008–2010. From 2020 onward, ADHD IRs rose sharply in both sexes, reaching 114 per 10,000 in males and 105 per 10,000 in females by 2024, accompanied by a marked narrowing of the sex gap in IRs (Fig. 1B). Overall incidence rates over time for ASD and ADHD can be found in Online Resource Fig. S1.

**Fig. 2:**
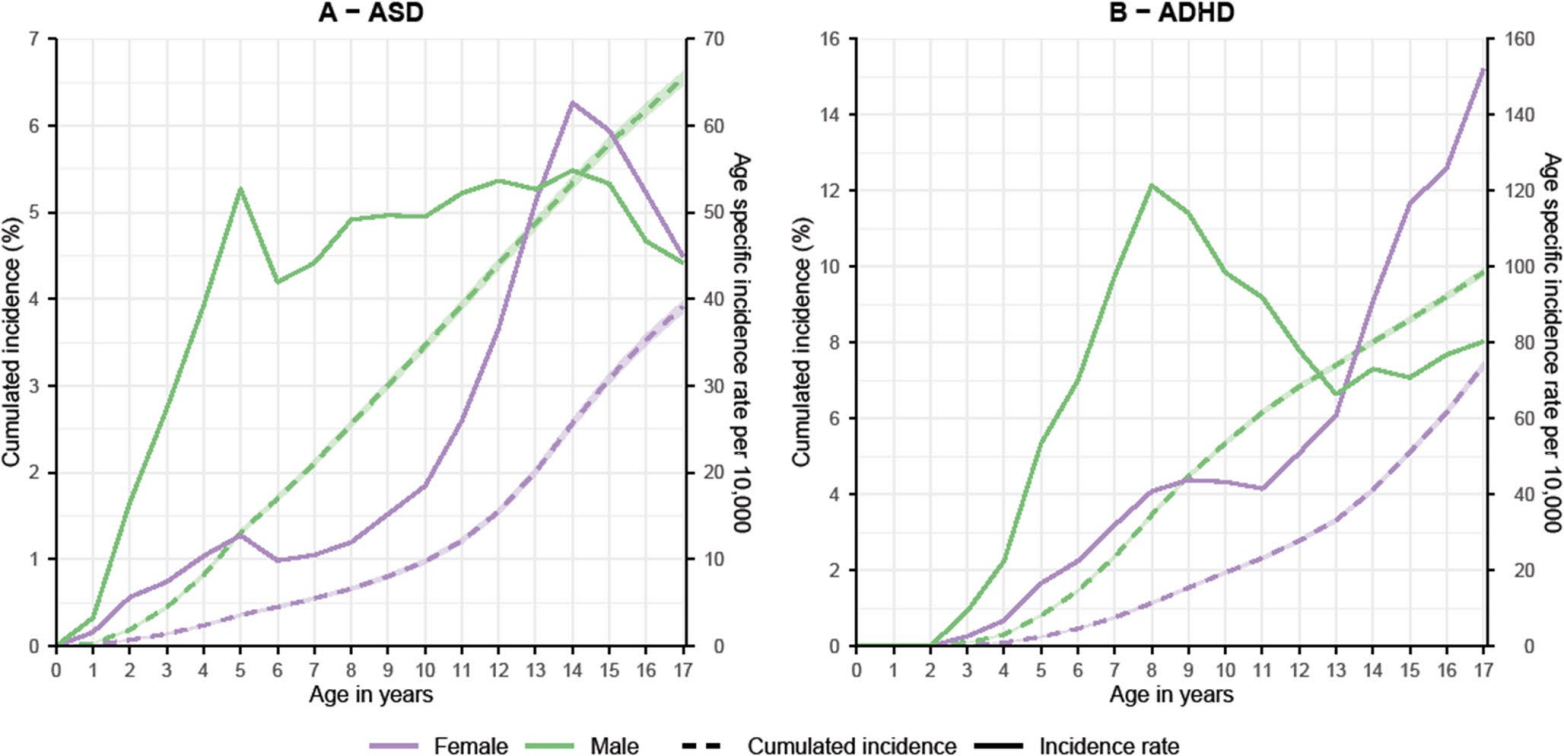
Incidence rates and age specific cumulative incidence proportions with 95% confidence intervals of (**A**) Autism Spectrum Disorders (ASD) and (**B**) Attention-Deficit/Hyperactivity Disorder (ADHD) according to sex in Danish children born from 2000 to 2023

Age-specific incidence rates varied by sex (Fig. 2A-B). For ASD, the IR peaked for males at age 5 and plateaued until age 15. In females, the IR remained low until age 8, although a smaller peak was observed at age 5, possibly reflecting cases of early-onset (infantile) autism, after which it increased steadily, peaking at age 14 (Fig. 2A). A similar pattern of higher age at diagnosis in females than males was observed for ADHD. In males, the IR peaked at age 8 and declined thereafter; in females, the IR reached a plateau at age 8 to 12, and then increased sharply until its highest level at age 17 (Fig. 2B). By age 18, the cumulative incidence of ASD was 6.5% (95% CI 6.5–6.6) in males and 3.9% (95% CI 3.8-4.0) in females; for ADHD, the corresponding estimates were 9.8% (95% CI 9.8–9.9) and 7.4% (95% CI 7.3–7.5) (Fig. 2A–B). Overall IR by age and cumulative incidence for ASD and ADHD can be found in Online Resource Fig. S2.

**Fig. 3:**
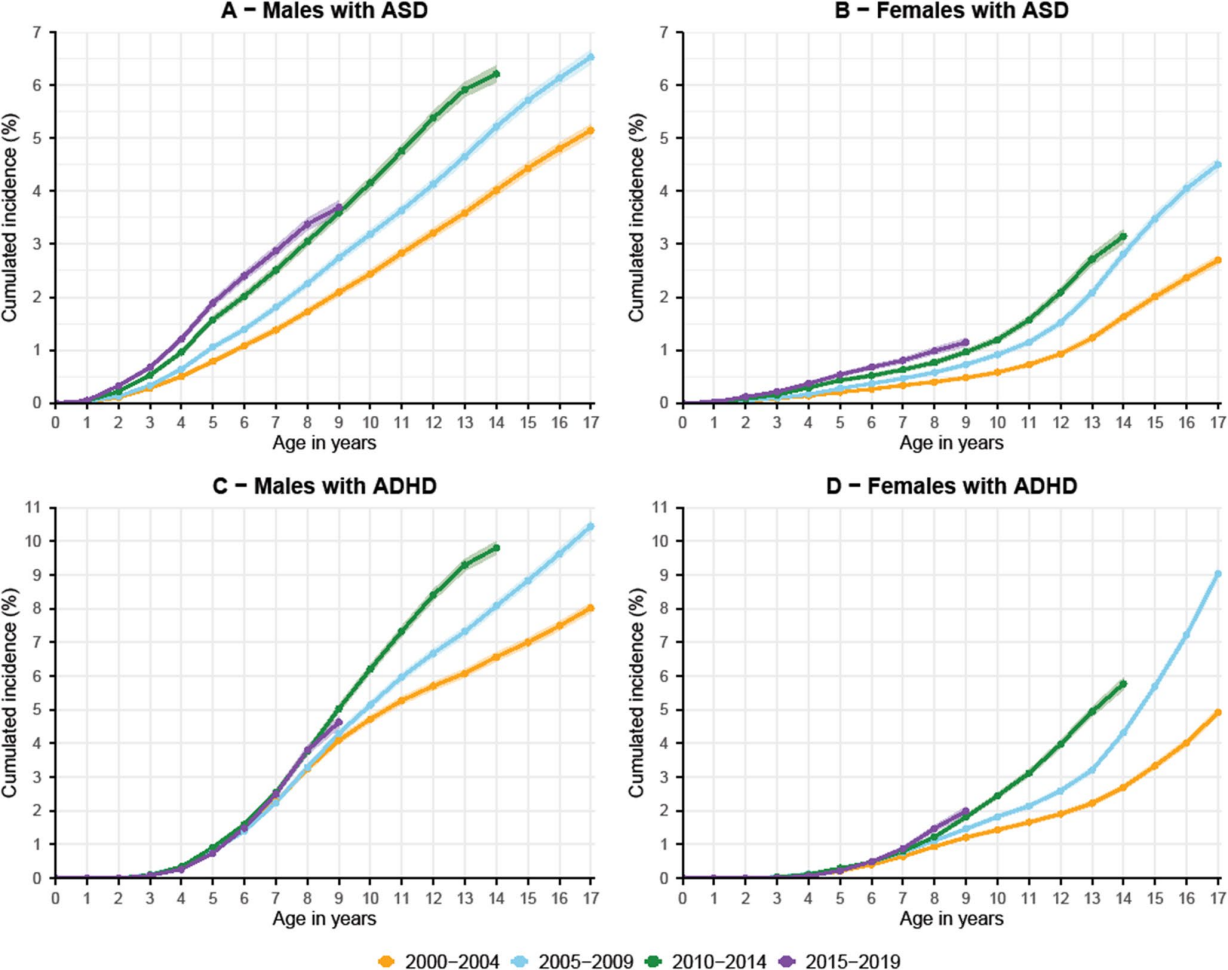
Cumulative incidence proportions with 95% confidence intervals of Autism Spectrum Disorders (ASD) by (**A**) males and (**B**) females, and of Attention-Deficit/Hyperactivity Disorder (ADHD) by (**C**) males and (**D**) females stratified into 5-year birth cohorts (2000–2004, 2005–2009, 2010–2014, and 2015–2019)

Cumulative incidences of both ASD and ADHD were higher in more recent birth cohorts for males and females (Fig. 3). Sensitivity analyses using stricter definitions of ASD and ADHD - requiring at least two recorded diagnoses of ASD, or either two diagnoses, two prescriptions of ADHD-specific medications, or one of each for ADHD – resulted in slightly lower cumulative incidence estimates (4.3% (95% CI 4.2–4.3) and 7.9% (95% CI 7.8-8.0) vs. 5.3% (95% CI 5.2–5.3) and 8.7% (95% CI 8.6–8.7), respectively). However, temporal trends and incidence patterns remained consistent with the main analyses (Online Resource Fig. S3-6). Exact age-specific IRs and cumulative incidence estimates are provided in Online Resource Tables S1-4.

**Fig. 4:**
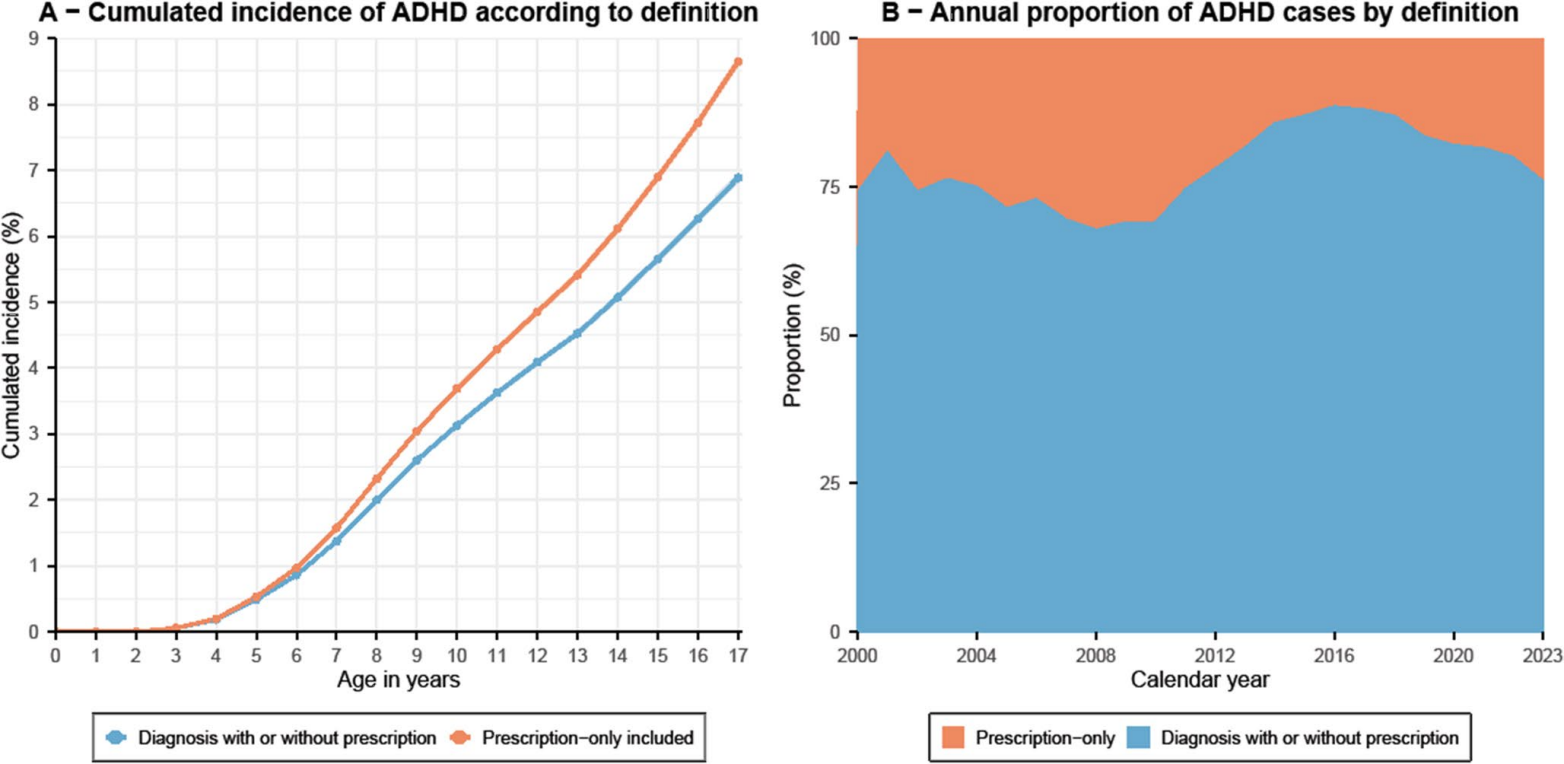
**A**. Cumulative incidence with 95% confidence intervals of ADHD by (1) diagnosis (ICD-10 F90 or F98.8) with or without prescription of an ADHD drug (methylphenidate, atomoxetine, dexamfetamine, lisdexamfetamine, or guanfacine) (blue) or (2) Definition 1 plus prescription only defined by filled prescription of an ADHD drug without any registration of a diagnosis within 365 days forward (orange). **B**. Annual proportions of ADHD cases defined only by a filled prescription (orange) and by diagnoses with or without prescription of an ADHD drug (blue)

Finally, we evaluated the impact of including individuals redeeming incident prescriptions for ADHD-specific medications in our definition of new ADHD cases. This expansion of the ADHD definition increased the cumulative incidence at age 18 years from 6.9% (95% CI 6.8–6.9) to 8.7% (95% CI 8.6–8.7), whereas the two groups overlapped until age 7 years (Fig. 4A). The proportion of ADHD cases identified solely through incident prescription redemptions varied over time, ranging from 11% in 2016 to 24% in 2023 (Fig. 4B).

## Discussion

In this nationwide Danish cohort study, we found a substantial increase in the incidence of ASD and ADHD over the past two decades, consistent with global trends. The rise was most pronounced for ADHD, particularly among females, who by 2024 had nearly reached the same IRs as males – narrowing a sex gap that was previously substantial. By age 18, 6.5% of males and 3.9% of females had been diagnosed with ASD, while 9.9% and 7.4% had received an ADHD diagnosis, respectively. The age at diagnosis for both ASD and ADHD changed throughout the study period towards a younger age at diagnosis in more recent birth cohorts. Using both prescription data and diagnosis codes from hospital-based care to define incident ADHD cases increased the cumulative incidence of ADHD from 6.9% to 8.7% by age 18. This suggests that relying solely on hospital diagnoses may underestimate the true incidence of individuals with ADHD, particularly among adolescents.

The main strength of our study was the use of nationwide health registers, enabling inclusion of an un-selected cohort of all children and adolescents in Denmark. Our definition of ADHD included ADHD medication use on top of hospital diagnoses, leading to improved case ascertainment and IR estimates. Our study also holds limitations. The Danish registers used in this study have high documented validity [21, 22, 26, 27], yet some degree of under-ascertainment and diagnostic misclassification is unavoidable in register-based data. In Denmark, an increasing number of ADHD and ASD assessments in children and adolescents take place outside hospital-based child and adolescent psychiatry in private psychiatric practice. Nevertheless, in 2024, 92% of registered ADHD diagnoses and 91% of registered ASD diagnoses were made in the hospital sector and thereby captured in our data [28]. As all redeemed prescriptions are recorded in the Danish National Prescription Registry, inclusion of ADHD-specific pharmacotherapy likely reduces under-ascertainment; however, individuals assessed and managed without medication may remain unidentified. Finally, while confounding is not applicable in this population-based descriptive study, future studies should aim to investigate determinants of observed trends including changes in diagnostic thresholds, referral patterns, and sociodemographic factors.

Overall, our findings align with prior studies documenting rising trends in both ASD and ADHD. The number of children diagnosed with ASD has increased steadily over recent decades in a number of countries, including Germany, the United Kingdom, and the United States [16, 29, 30]. Globally, ASD prevalence varies widely (0.1%-4.4%) across regions, likely reflecting methodological differences in measurements of ASD prevalence as well as variation in community awareness, diagnostic practices, and access to care [31]. Our estimated cumulative incidence of ASD (6.5% in males and 3.9% in females) exceeds the upper range of these global findings as well as previous Danish estimates [4]. In line with existing research, we observed a consistently higher incidence of ASD in males [16, 31, 32]. While some studies have reported a relatively steeper increase in ASD among females, leading to a decreasing male-to-female ratio [16, 32], our findings indicate parallel increases across sexes, although girls are diagnosed at a substantially older age than boys.

The incidence of clinically diagnosed ADHD has also increased in past decades, reaching global prevalence estimates of 8.0% among children and adolescents [33, 34]. Our finding that nearly 10% of boys and 7% of girls are diagnosed with ADHD by age 18, with indications of even higher prevalence in more recent birth cohorts, is notable. Several factors likely contribute to this trend, including improved access to treatment and growing awareness of sex-specific manifestations of ADHD, particularly the increased recognition of ADHD in females. Additional contributing factors include increased knowledge and awareness of ADHD among health care providers, parents, and educators [35]. The use of psychostimulant medications for ADHD has risen substantially since 2020 [9, 19, 36]. While this has partly been linked to pandemic-related stress and social restrictions [15, 37], our data show that the increase in ADHD incidence began prior to the pandemic and has continued thereafter, suggesting broader underlying drivers that warrant further investigation.

Internationally, ADHD prevalence has been reported to be approximately twice as high in boys (10%) as in girls (5%) [33], with evidence indicating that ADHD in females is often later recognized and treated [38, 39]. In our study, this sex disparity was less pronounced, with 9.8% of boys and 7.4% of girls diagnosed before age 18. Notably, from 2022 onwards, the incidence in girls nearly equaled that of boys, potentially suggesting improved recognition of ADHD among females in Denmark.

We evaluated the impact of combining both diagnostic data and redeemed prescriptions to define new cases of ADHD, which increased the estimated cumulative incidence proportion by age 18 year by 20%. This finding highlights the potential underestimation inherent in diagnosis-only definitions in register-based studies. The divergence at older ages likely reflects increasing use of private specialist assessments, where diagnoses are not recorded but prescriptions are captured. As waiting times for hospital-based child and adolescent psychiatric services increase, more families may seek evaluation and help in private settings, either through publicly subsidized providers or private insurance. This is supported by our finding of a growing discrepancy between diagnosis-based and prescription-only based ADHD definitions in recent years.

Our findings confirm that an increasing proportion of Danish children and adolescents are diagnosed with neurodevelopmental disorders, most notably young females with ADHD. Mechanisms driving this development are likely multifaceted and remain only partially understood. Possible contributors include increased public and clinical awareness, greater recognition of ADHD in girls, and changes in help-seeking and referral patterns during and after the COVID-19 period [14, 15, 40]. Greater awareness may lead to increased help-seeking and assessment of children with a broader range of symptom severity. In combination with constraints in service capacity and increased expectations for clinical support, this may in turn influence diagnostic practices [41]. With nearly 10% of children and adolescents diagnosed with ADHD and 5% with ASD in more recent birth cohorts, we need to consider whether these increases reflect improved diagnostic practices and recognition of previously undiagnosed individuals, or diagnostic inflation leading to diagnosis and medical treatment in children and adolescents with ambiguous or milder symptoms [42]. It is also possible that the Danish school system reforms in 2012 and 2013 that, respectively, introduced broader inclusion criteria for children with special needs and longer and less uniform school days, which may have worsened neurodevelopmental symptoms in some children leading to an increased demand for diagnostic clarification and potential medical treatment [43, 44]. This is particularly important as long-term safety data of the most used psychostimulant medication methylphenidate, remain limited and the association with adverse effects is uncertain [45].

In this nationwide Danish cohort, the incidence of both ASD and ADHD increased substantially over the study period, with marked temporal and sex-specific variations. While boys were diagnosed earlier and more frequently in early childhood, girls experienced a later but substantial surge in diagnoses in adolescence, effectively narrowing the historical sex gap for ADHD. This convergence suggests improved recognition of neurodevelopmental disorders, particularly in females, but may also reflect shifts in help-seeking behavior and diagnostic pathways. The inclusion of prescription data substantially increased incidence estimates, underscoring the importance of using broader case definitions in epidemiological research. The high proportion of Danish children diagnosed with ASD and ADHD highlights the importance of reflecting on current practices, including the widespread use of medication with limited knowledge of long-term side effects, and the need to consider non-pharmacological interventions.

## Supplementary Information

Below is the link to the electronic supplementary material.

Supplementary Material 1 (DOCX 682 KB)

## Data Availability

The data that support the finding of this study are not openly available due to reasons of sensitivity. Data are located at secure serves at the Danish Health Data Authorities and can be applied for under Danish legislation. R scripts for data analysis are available from the corresponding author upon request.
