# Supplementary material for: Trends and incidence rates of neurodevelopmental disorders in Danish children and adolescents 2000–2024: Short title: neurodevelopmental disorders in children and adolescents
Source: Eur Child Adolesc Psychiatry. 2026 Feb 10;35(5):1667–75. doi: 10.1007/s00787-026-02981-0 (PMC13272209; doi:10.1007/s00787-026-02981-0)
Supplement: Supplementary file 1 — Supplementary Material 1 (DOCX 682 KB) [file 787_2026_2981_MOESM1_ESM.docx]

**Trends and incidence rates of neurodevelopmental disorders in Danish children and adolescents**

Journal: European Child and Adolescent Psychiatry

Authors: Bliddal, M, Gram EB, Sonne H, Krumborg JR, Lundgaard KM, Wesselhoeft R, Kildegaard, H.

Corresponding author:

Mette Bliddal

University of Southern Denmark

E-mail: mbliddal@health.sdu.dk

**Supplementary Tables and Figures**

Table S1. Annual incidence rates (IRs) of Autism spectrum disorders (ASD) per 10,000 individuals, overall and stratified by sex

| **All** | | | |  | **Female** | | | |  | **Male** | | | |
| --- | --- | --- | --- | --- | --- | --- | --- | --- | --- | --- | --- | --- | --- |
| **Age (years)** | **Total** | **ASD** | **IR/10,000** |  | **Age (years)** | **Total** | **ASD** | **IR/10,000** |  | **Age (years)** | **Total** | **ASD** | **IR/10,000** |
| 0 | 1,468,235 | 0 | 0 |  | 0 | 714,740 | 0 | 0 |  | 0 | 753,495 | 0 | 0 |
| 1 | 1,399,564 | 342 | 2.4 |  | 1 | 681,440 | 108 | 1.6 |  | 1 | 718,124 | 234 | 3.3 |
| 2 | 1,332,166 | 1,498 | 11 |  | 2 | 649,137 | 366 | 5.6 |  | 2 | 683,029 | 1,132 | 17 |
| 3 | 1,261,166 | 2,222 | 18 |  | 3 | 615,194 | 455 | 7.4 |  | 3 | 645,972 | 1,767 | 27 |
| 4 | 1,193,763 | 3,003 | 25 |  | 4 | 583,121 | 605 | 10 |  | 4 | 610,642 | 2,398 | 39 |
| 5 | 1,127,035 | 3,736 | 33 |  | 5 | 551,659 | 704 | 13 |  | 5 | 575,376 | 3,032 | 53 |
| 6 | 1,062,674 | 2,787 | 26 |  | 6 | 520,953 | 513 | 10 |  | 6 | 541,721 | 2,274 | 42 |
| 7 | 999,623 | 2,761 | 28 |  | 7 | 490,754 | 515 | 10 |  | 7 | 508,869 | 2,246 | 44 |
| 8 | 937,012 | 2,891 | 31 |  | 8 | 460,899 | 551 | 12 |  | 8 | 476,113 | 2,340 | 49 |
| 9 | 878,297 | 2,871 | 33 |  | 9 | 432,660 | 658 | 15 |  | 9 | 445,637 | 2,213 | 50 |
| 10 | 821,481 | 2,807 | 34 |  | 10 | 405,225 | 747 | 18 |  | 10 | 416,256 | 2,060 | 49 |
| 11 | 765,731 | 3,004 | 39 |  | 11 | 378,089 | 981 | 26 |  | 11 | 387,642 | 2,023 | 52 |
| 12 | 708,554 | 3,205 | 45 |  | 12 | 350,122 | 1,282 | 37 |  | 12 | 358,432 | 1,923 | 54 |
| 13 | 650,700 | 3,379 | 52 |  | 13 | 321,242 | 1,644 | 51 |  | 13 | 329,458 | 1,735 | 53 |
| 14 | 589,349 | 3,457 | 59 |  | 14 | 290,752 | 1,820 | 63 |  | 14 | 298,597 | 1,637 | 55 |
| 15 | 529,136 | 2,980 | 56 |  | 15 | 261,014 | 1,551 | 59 |  | 15 | 268,122 | 1,429 | 53 |
| 16 | 467,313 | 2,309 | 49 |  | 16 | 230,546 | 1,204 | 52 |  | 16 | 236,767 | 1,105 | 47 |
| 17 | 407,657 | 1,815 | 45 |  | 17 | 201,133 | 903 | 45 |  | 17 | 206,524 | 912 | 44 |

Table S2. Annual incidence rates (IRs) of Attention-Deficit/Hyperactivity Disorder (ADHD) per 10,000 individuals, overall and stratified by sex

| **Age (years)** | **Total** | **ADHD** | **IR/10,000** |  | **Age (years)** | **Total** | **ADHD** | **IR/10,000** |  | **Age (years)** | **Total** | **ADHD** | **IR/10,000** |
| --- | --- | --- | --- | --- | --- | --- | --- | --- | --- | --- | --- | --- | --- |
| 0 | 1,468,235 | 0 | 0 |  | 0 | 714,740 | 0 | 0 |  | 0 | 753,495 | 0 | 0 |
| 1 | 1,399,902 | 0 | 0 |  | 1 | 681,548 | 0 | 0 |  | 1 | 718,354 | 0 | 0 |
| 2 | 1,333,953 | 0 | 0 |  | 2 | 649,599 | 0 | 0 |  | 2 | 684,354 | 0 | 0 |
| 3 | 1,264,238 | 752 | 5,9 |  | 3 | 615,901 | 158 | 2,6 |  | 3 | 648,337 | 594 | 9.2 |
| 4 | 1,197,680 | 1,770 | 15 |  | 4 | 583,945 | 392 | 6.7 |  | 4 | 613,735 | 1,378 | 22 |
| 5 | 1,130,124 | 3,997 | 35 |  | 5 | 552,134 | 912 | 17 |  | 5 | 577,990 | 3,085 | 53 |
| 6 | 1,063,093 | 4,963 | 47 |  | 6 | 520,693 | 1,166 | 22 |  | 6 | 542,400 | 3,797 | 70 |
| 7 | 996,132 | 6,506 | 65 |  | 7 | 489,437 | 1,561 | 32 |  | 7 | 506,695 | 4,945 | 98 |
| 8 | 929,129 | 7,580 | 82 |  | 8 | 458,400 | 1,868 | 41 |  | 8 | 470,729 | 5,712 | 121 |
| 9 | 867,196 | 6,874 | 79 |  | 9 | 429,268 | 1,879 | 44 |  | 9 | 437,928 | 4,995 | 114 |
| 10 | 808,418 | 5,742 | 71 |  | 10 | 401,205 | 1,735 | 43 |  | 10 | 407,213 | 4,007 | 98 |
| 11 | 751,932 | 5,024 | 67 |  | 11 | 373,982 | 1,552 | 41 |  | 11 | 377,950 | 3,472 | 92 |
| 12 | 694,851 | 4,478 | 64 |  | 12 | 346,113 | 1,759 | 51 |  | 12 | 348,738 | 2,719 | 78 |
| 13 | 637,824 | 4,057 | 64 |  | 13 | 317,487 | 1,933 | 61 |  | 13 | 320,337 | 2,124 | 66 |
| 14 | 576,993 | 4,723 | 82 |  | 14 | 286,906 | 2,605 | 91 |  | 14 | 290,087 | 2,118 | 73 |
| 15 | 516,962 | 4,831 | 93 |  | 15 | 256,561 | 2,989 | 117 |  | 15 | 260,401 | 1,842 | 71 |
| 16 | 455,046 | 4,603 | 101 |  | 16 | 225,514 | 2,841 | 126 |  | 16 | 229,532 | 1,762 | 77 |
| 17 | 395,189 | 4,573 | 116 |  | 17 | 195,345 | 2,969 | 152 |  | 17 | 199,844 | 1,604 | 80 |

Table S3. Cumulative incidence with 95% confidence intervals of autism spectrum disorders per 10,000 individuals, overall and stratified by sex

| **All** | |  | **Female** | |  | **Male** | |
| --- | --- | --- | --- | --- | --- | --- | --- |
| **Age (years)** | **Cumulative incidence/10,000** |  | **Age (years)** | **Cumulative incidence/10,000** |  | **Age (years)** | **Cumulative incidence/10,000** |
| 0 | 0.00 (0.00-0.00) |  | 0 | 0.00 (0.00-0.00) |  | 0 | 0.00 (0.00-0.00) |
| 1 | 0.02 (0.02 -0.03) |  | 1 | 0.02 (0.01-0.02) |  | 1 | 0.03 (0.03-0.04) |
| 2 | 0.13 (0.12-0.14) |  | 2 | 0.07 (0.06-0.08) |  | 2 | 0.19 (0.18-020) |
| 3 | 0.30 (0.29-0.31) |  | 3 | 0.14 (0.13-0.15) |  | 3 | 0.45 (0.43-0.46) |
| 4 | 0.53 (0.52-055) |  | 4 | 0.24 (0.23-0.25) |  | 4 | 0.82 (0.79-0.84) |
| 5 | 0.85 (0.83- 0.86) |  | 5 | 0.36 (0.34-0.37) |  | 5 | 1.3 (1.3-1.3) |
| 6 | 1.1 (1.1-1.1) |  | 6 | 0.45 (0.43-0.47) |  | 6 | 1.7 (1.7-1.7) |
| 7 | 1.3 (1.3-1.4) |  | 7 | 0.55 (0.53-0.57) |  | 7 | 2.1 (2.1-2.1) |
| 8 | 1.6 (1.6-1.7) |  | 8 | 0.66 (0.64-0.68) |  | 8 | 2.6 (2.5-2.6) |
| 9 | 1.9 (1.9-2.0) |  | 9 | 0.80 (0.78-0.83) |  | 9 | 3.0 (3.0-3.1) |
| 10 | 2.2 (2.2-2.3) |  | 10 | 0.97 (0.95-1.00) |  | 10 | 3.5 (3.4-3.5) |
| 11 | 2.6 (2.6-2.6) |  | 11 | 1.2 (1.2-1.2) |  | 11 | 3.9 (3.9-4.0) |
| 12 | 3.0 (3.0-3.0) |  | 12 | 1.5 (1.5-1.6) |  | 12 | 4.4 (4.3-4.5) |
| 13 | 3.5 (3.4-3.5) |  | 13 | 2.0 (2.0-2.1) |  | 13 | 4.9 (4.8-4.9) |
| 14 | 4.0 (3.9-4.0) |  | 14 | 2.6 (2.5-2.6) |  | 14 | 5.3 (5.3-5.3) |
| 15 | 4.5 (4.4-4.5) |  | 15 | 3.1 (3.0-3.1) |  | 15 | 5.8 (5.7-5.9) |
| 16 | 4.9 (4.8-4.9) |  | 16 | 3.5 (3.5-3.6) |  | 16 | 6.2 (6.1-6.3) |
| 17 | 5.3 (5.2-5.3) |  | 17 | 3.9 (3.8-4.0) |  | 17 | 6.5 (6.5-6.6) |

Table S4. Cumulative incidence (95% confidence intervals) of Attention-Deficit/Hyperactivity Disorder per 10,000 individuals, overall and stratified by sex

| **All** | |  | **Female** | |  | **Male** | |
| --- | --- | --- | --- | --- | --- | --- | --- |
| **Age (years)** | **Cumulative incidence/10,000** |  | **Age (years)** | **Cumulative incidence/10,000** |  | **Age (years)** | **Cumulative incidence/10,000** |
| 0 | 0.00 (0.00-0.00) |  | 0 | 0.00 (0.00-0.00) |  | 0 | 0.00 (0.00-0.00) |
| 1 | 0.00 (0.00-0.00) |  | 1 | 0.00 (0.00-0.00) |  | 1 | 0.00 (0.00-0.00) |
| 2 | 0.00 (0.00-0.00) |  | 2 | 0.00 (0.00-0.00) |  | 2 | 0.00 (0.00-0.00) |
| 3 | 0.06 (0.05-0.06) |  | 3 | 0.02 (0.02-0.03) |  | 3 | 0.09 (0.08-0.09) |
| 4 | 0.20 (0.19-0.20) |  | 4 | 0.09 (0.08-0.10) |  | 4 | 0.30 (0.29-0.31) |
| 5 | 0.53 (0.52-0.54) |  | 5 | 0.24 (0.23-0.26) |  | 5 | 0.80 (0.78-0.82) |
| 6 | 0.97 (0.95-0.98) |  | 6 | 0.45 (0.44-0.47) |  | 6 | 1.5 (1.4-1.5) |
| 7 | 1.6 (1.5-1.6) |  | 7 | 0.75 (0.73-0.78) |  | 7 | 2.4 (2.3-2.4) |
| 8 | 2.3 (2.3-2.3) |  | 8 | 1.1 (1.2-1.2) |  | 8 | 3.5 (3.4-3.5) |
| 9 | 3.0 (3.0-3.1) |  | 9 | 1.5 (1.5-1.6) |  | 9 | 4.5 (4.4-4.5) |
| 10 | 3.7 (3.6-3.7) |  | 10 | 1.9 (1.9-2.0) |  | 10 | 5.3 (5.3-5.4) |
| 11 | 4.3 (4.2-4.3) |  | 11 | 2.3 (2.3-2.4) |  | 11 | 6.2 (6.1-6.2) |
| 12 | 4.9 (4.8-4.9) |  | 12 | 2.8 (2.7-2.8) |  | 12 | 6.8 (6.8-6.9) |
| 13 | 5.4 (5.4-5.5) |  | 13 | 3.3 (3.3-3.4) |  | 13 | 7.4 (7.3-7.5) |
| 14 | 6.1 (6.1-6.2) |  | 14 | 4.1 (4.0-4.2) |  | 14 | 8.0 (7.9-8.1) |
| 15 | 6.9 (6.8-7.0) |  | 15 | 5.1 (5.0-5.2) |  | 15 | 8.6 (8.5-8.7) |
| 16 | 7.7 (7.7-7.8) |  | 16 | 6.2 (6.1-6.2) |  | 16 | 9.2 (9.1-9.3) |
| 17 | 8.7 (8.6-8.7) |  | 17 | 7.4 (7.3-7.5) |  | 17 | 9.8 (9.8-9.9) |


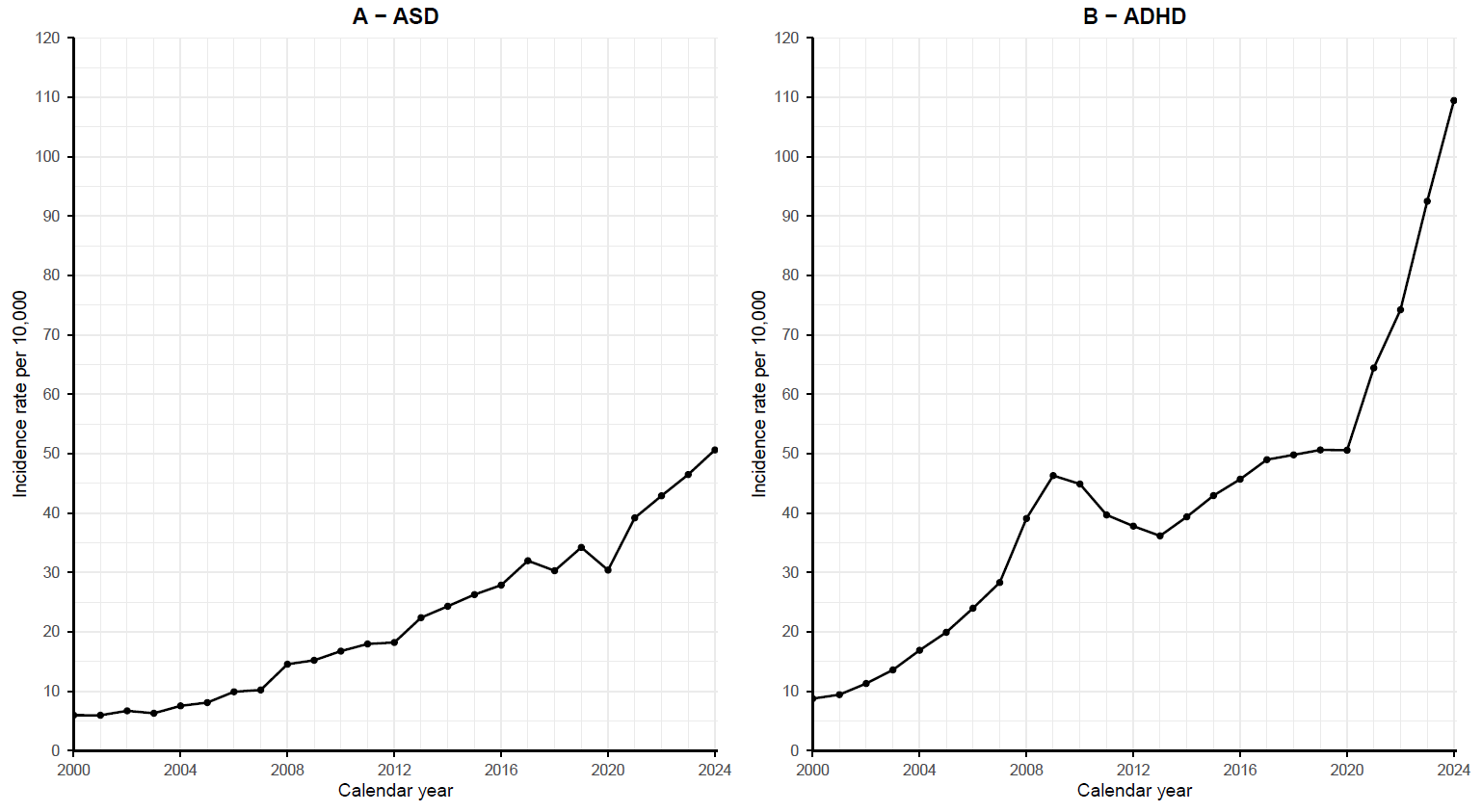


**Fig. S1** Overall incidence rates of A) Autism Spectrum Disorders (ASD) and B) Attention-Deficit/Hyperactivity Disorder (ADHD) in Danish children aged 0-17 years per 10,000 person years from 2000 to 2024.

**Fig. S2** Overall incidence rates and age specific cumulative incidence proportions (95% confidence intervals) of A) Autism Spectrum Disorders (ADS) and B) Attention-Deficit/Hyperactivity Disorder (ADHD) in Danish children born from 2000 to 2023.


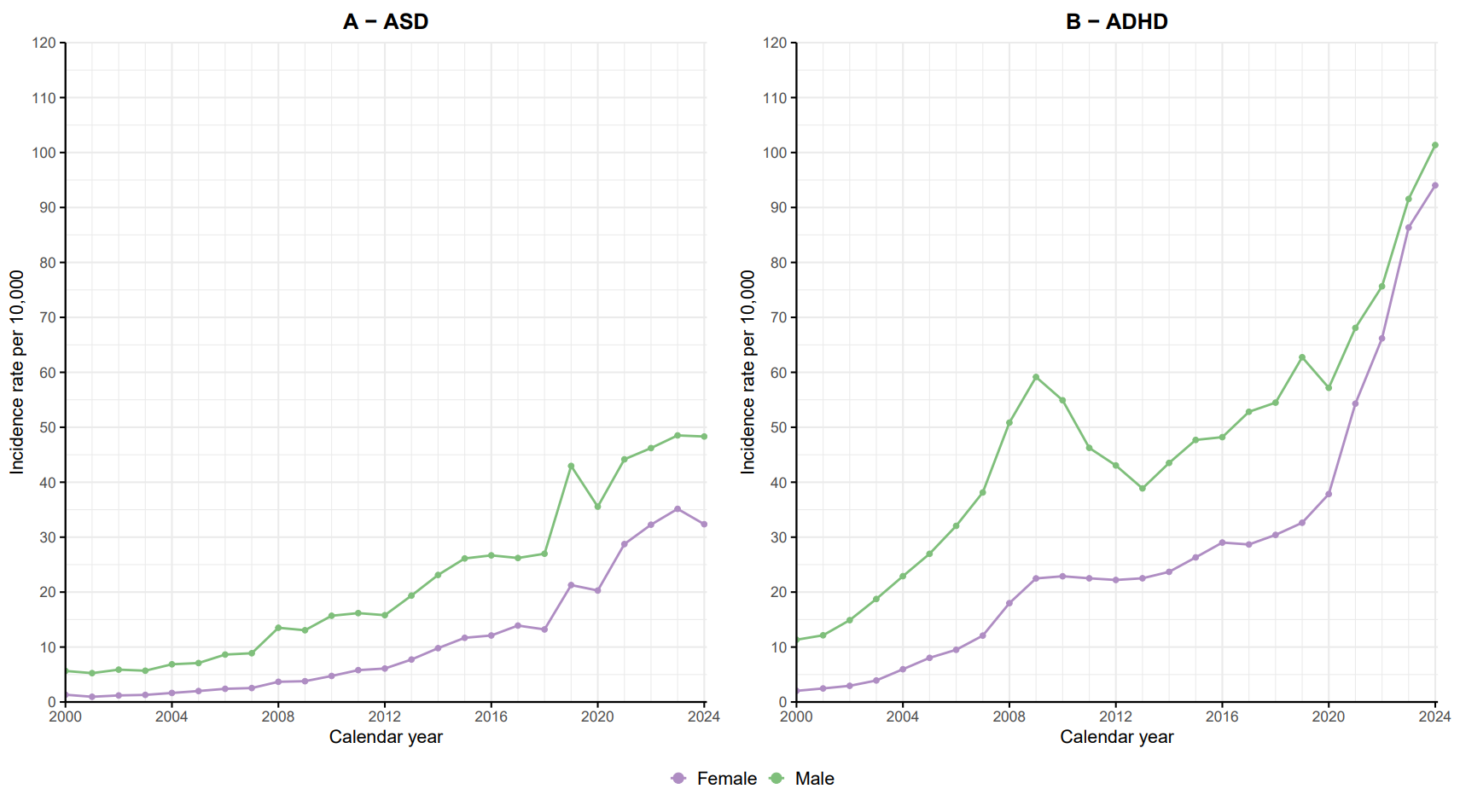


**Fig. S3** Incidence rates of A) autism spectrum disorders (ASD) (ICD10 F84) and B) ADHD (F90 and F98.8) in Danish children aged 0-17 years per 1,000 person years from 2000 to 2023***, restricted to at least two markers of the respective disorders.***

**Fig. S4** Incidence rates and age specific cumulative incidence proportions (95% confidence intervals) of A) Autism Spectrum Disorders (ADS) and B) Attention-Deficit/Hyperactivity Disorder (ADHD) according to sex in Danish children born from 2000 to 2023***, restricted to at least two markers of the respective disorders***.

**Fig. S5** Cumulative incidence proportions (95% confidence intervals) of Autism Spectrum Disorders (ASD) by A) males and B) female, and of Attention-Deficit/Hyperactivity Disorder (ADHD) by C) males and D) females stratified into 5-year birth cohorts (2000-2004, 2005-2009, 2010-2014, and 2015-2019). ***Restricted to at least two markers of the respective disorders.***

**Fig. S6** A. Cumulative incidence (95% confidence intervals) of ADHD by 1) diagnosis (ICD-10 F90 or F98.8) with or without prescription of an ADHD drug (methylphenidate, atomoxetine, dexamfetamine, lisdexamfetamine, or guanfacine) (blue) or 2) Definion 1 plus prescription only defined by filled prescription of an ADHD drug without any registration of a diagnosis within 365 days forward (orange). B. Annual proportions of ADHD cases defined only by a filled prescription (orange) and by diagnoses with or without prescription of an ADHD drug (blue). ***Restricted to at least two markers of the respective disorders per outcome definition.***
